# Hemorrhagic Cardiac Tamponade as a Complication of Limited Cutaneous Systemic Sclerosis

**DOI:** 10.7759/cureus.39947

**Published:** 2023-06-04

**Authors:** Faria Sami, Shahzad Ahmed Sami, Saman Tanveer, Hania Sami

**Affiliations:** 1 Internal Medicine, Allama Iqbal Medical College, Lahore, PAK; 2 Internal Medicine, Trinity Health Oakland Hospital, Pontiac, USA; 3 Internal Medicine, Army Medical College, National University of Medical Sciences (NUMS), Rawalpindi, PAK; 4 Anatomy, Physiology, and Biochemistry, Shalamar Medical and Dental College, Lahore, PAK

**Keywords:** pericardial effusion, limited cutaneous systemic sclerosis, tamponade, systemic sclerosis, scleroderma

## Abstract

Cardiac tamponade is an uncommon complication of systemic sclerosis (SSc) with a high mortality rate. Here, we report a case of a 58-year-old patient with limited cutaneous systemic sclerosis (lcSSc), gastroesophageal reflux disease (GERD), diabetes mellitus, pulmonary hypertension (PHTN), and COVID-19 infection, which occurred one month ago, presenting with a large hemorrhagic pericardial effusion and early cardiac tamponade. The patient had an acute onset of progressive dyspnea and anasarca. On examination, she was tachypneic, tachycardic, desaturating on room air, and hypotensive. Pitting edema up to thighs and bilateral basilar crackles were also appreciated. Labs were remarkable for negative troponin, chest X-ray with pulmonary congestion, D-dimer at 6.01, CT angiogram negative, brain natriuretic peptide level at 73 pg/mL, C-reactive protein level at 7.64 mg/dL, normal complement levels, and negative COVID-19 test results. Echocardiography showed early tamponade and a large circumferential effusion with chamber collapse. Right heart catheterization was performed finding PHTN at 54 mmHg. Pericardiocentesis drained 500 mL of the hemorrhagic effusion. Fluid analysis showed RBC at 220,000/uL, WBC at 5000/uL, protein 4.8 g/dL, lactate dehydrogenase level of 1275 U/L, and negative cytology. The patient was treated for serositis from lcSSc flare with mycophenolate mofetil and steroids, and responded very well. Hemorrhagic cardiac tamponade is a very rare phenomenon in limited scleroderma. A recent COVID-19 infection could have served as a trigger factor for our patient’s lcSSc in long remission to flare up. Clinicians should maintain a high index of suspicion and a low threshold for intervention when lcSSc patients have an acute onset of cardiac compromise, especially with a history of a recent COVID-19 infection.

## Introduction

Cardiac involvement in systemic sclerosis (SSc) is common with about 60% of patients with evidence of pericardial disease. Involvement is usually benign and silent with only a minority of patients becoming clinically symptomatic [1]. Although pericardial effusions are common in diffuse scleroderma, they are not a very common finding in limited cutaneous systemic sclerosis (lcSSc). Cardiac tamponade is an extremely rare and serious complication of pericardial effusion with a high mortality rate [2]. The fluid profile is usually exudative, and hemorrhagic effusions are not common. Pericardial effusions with tamponade morphology in SSc patients are often associated with poorer prognosis and higher mortality rates. We report an interesting case of a patient with lcSSc in remission presenting with a large hemorrhagic pericardial effusion and early cardiac tamponade. This case illustrates the importance of early diagnosis and management by keeping a high suspicion of tamponade in at-risk lcSSc patients.

## Case presentation

A 58-year-old Hispanic female with type 2 diabetes mellitus, gastroesophageal reflux disease (GERD), lcSSc, and pulmonary hypertension (PHTN) presented to our tertiary care center for the evaluation of the acute onset of progressively worsening dyspnea and lower extremity edema over the past week. She started to notice an acute onset of bilateral lower extremity edema, periorbital swelling, orthopnea, and dyspnea on exertion as well as rest. She denied fever, cough, chest pain, palpitations, dizziness, syncope, or new medications. She had a COVID-19 infection one month ago that had resolved without complications within one week. Her lcSSC was diagnosed seven years ago, did not require immunosuppressive treatment, and remained stable. GERD was diagnosed on endoscopy at the same time, with intermittent symptoms only. She also had echocardiography done at the time of diagnosis with pulmonary artery pressure of 40 mmHg and right ventricular dilatation but was not limited by symptoms.

On examination, she was found to be tachypneic, tachycardic, hypotensive (90/70 mmHg), and saturating 88% on room air. Pitting edema up to the thighs and periorbital swelling were found, and bilateral basilar lung crackles were auscultated. The patient was in distress and bending forward to improve dyspnea. Initial labs only revealed negative troponin, D-dimer elevated at 6.01, chest X-ray with diffuse pulmonary vascular congestion, brain natriuretic peptide level at 73 pg/mL, C-reactive protein 7.64 mg/dL, normal complement levels, and a negative COVID-19 PCR test (Table 1).

**Table 1:** Initial laboratory values BNP, brain natriuretic peptide; CRP, C-reactive protein

Lab	Patient's results	Reference range
D-dimer	6.01	0.27–0.49
BNP	73 pg/mL	<100 pg/mL
CRP	7.64 mg/dL	0.00–0.50 mg/dL
C3 complement	120 mg/dL	88–201 mg/dL
C4 Complement	18 mg/dL	16–47 mg/dL

**Figure 1:**
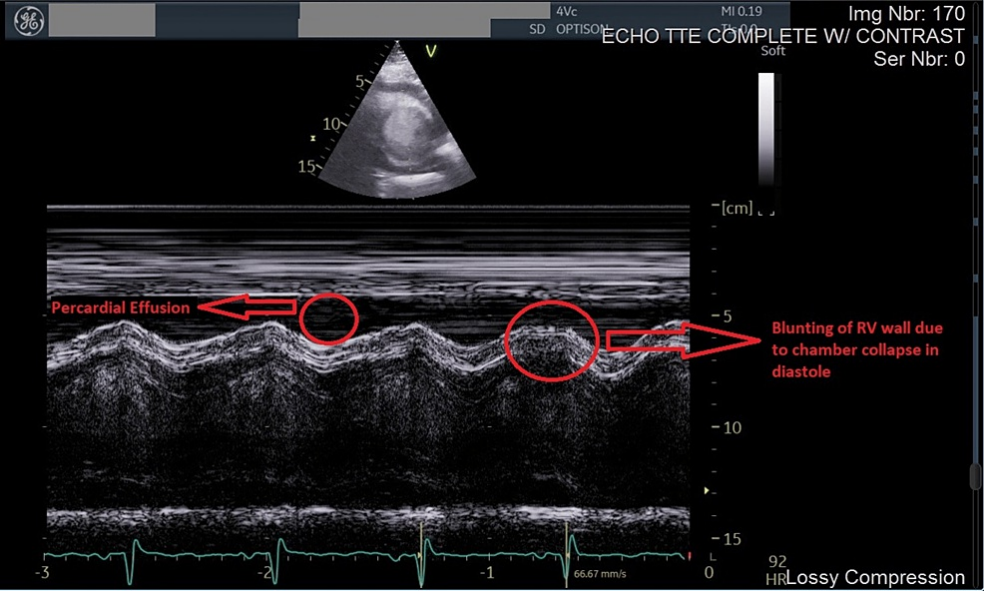
Right ventricular (RV) wall collapse during diastole on echocardiography

**Figure 2:**
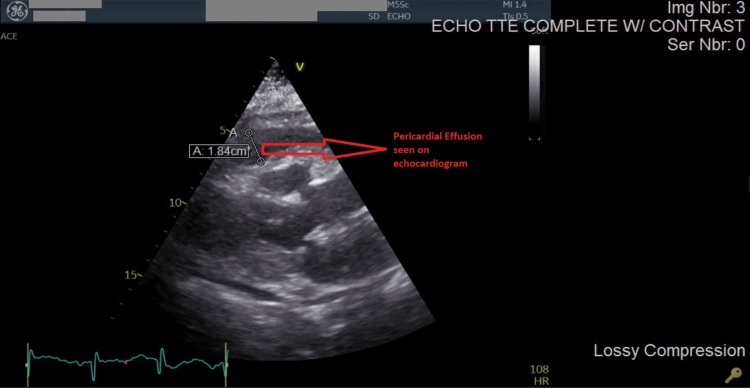
Pericardial effusion on echocardiographic imaging

The chest computed tomography (CT) angiogram was negative for pulmonary embolism. EKG showed normal sinus rhythm only and bedside echocardiography revealed a large pericardial effusion in the emergency department. Formal echocardiography showed tamponade physiology on Doppler imaging due to a massive circumferential pericardial effusion with chamber collapse, inferior vena cava dilatation, septal bounce, pulmonary artery pressure of 45 mmHg, and an ejection fraction of 50% (Figures 1, 2).

The patient underwent right heart catheterization with pulmonary artery pressure at 54 mmHg and wedge pressure at 51 mmHg. Nontraumatic pericardiocentesis drained grossly hemorrhagic 500 mL of the effusion. Peri-procedurally, the patient developed paroxysmal atrial fibrillation with a rapid ventricular response with spontaneous resolution. Fluid analysis showed RBC at 220,000/uL, WBC at 5000/uL (polymorphonuclear predominant), protein 4.8 g/dL, lactate dehydrogenase level of 1275 U/L, and negative cytology. Rheumatology service was consulted for concern of serositis in the setting of lcSSc. The patient was started on 40 mg of prednisone daily with a tapering regimen and drastically improved within two days with the resolution of symptoms and exam findings. The RNA polymerase-III test was ordered to assess for the risk of scleroderma renal crisis with steroid use. However, in light of the risk/benefit discussion, our patient with PHTN and tamponade was at risk of decompensation and was considered a candidate for steroid treatment. The patient was discharged to follow up at the clinic. The patient, on her last follow-up, had no acute symptoms and continued to do well on mycophenolate mofetil and low-dose prednisone.

## Discussion

Visceral involvement in patients with diffuse scleroderma is more frequent, severe, and early in onset, whereas it is the opposite for limited SSc [3]. Scleroderma cardiac complications can be variable from myocardial fibrosis, pericardial or valvular disease, to even arrhythmias. As mentioned earlier, immune-mediated inflammation of the pericardium commonly causes pericardial effusion in diffused systemic sclerosis. However, it is not a very common finding in limited cutaneous SSc [4]. SSc associated with large pericardial effusions (>200 mL) is associated with a poorer prognosis [4]. These effusions are exudative and tamponade is an uncommon presentation.

We reviewed the literature for cardiac tamponade with lcSSc. Sattar et al. reported a case of clinically significant, rapid progression of a moderate pericardial effusion to fatal tamponade in a 45-year-old patient with a 10-year history of lcSSc who also had PHTN [5]. Similarly, Yebra et al. described a case of a 57-year-old female with a 16-year history of lcSSc that presented with fevers, chest pain, and friction rub tamponade and recovered without known recurrence [6]. Nabatian et al. reported a case of recurrent pericardial effusion with tamponade in a lcSSc patient without pulmonary or renal involvement, who eventually got a pericardial window [7]. Plotnick et al. reported a case of a lcSSc patient with pericardial effusion causing hemodynamic compromise (drained 350 cc), without evidence of right heart collapse and pulmonary artery pressure of 24 mmHg [8].

Several studies have reported a poor prognosis and a higher mortality rate from pericardial effusions with tamponade morphology in SSc patients. Outcomes are worse, especially in the presence of PHTN and renal disease, which are the two most common comorbidities observed in these patients as noted from the literature reported [9]. Patients with PHTN in lcSSc who develop pericardial effusions, especially, have higher mortality rates. For these patients, lowering pulmonary pressures may be necessary before any intervention. In our patient, given the high-risk presentation, we proceeded to measure pulmonary pressures through right heart catheterization followed by pericardiocentesis to consider the need for optimization prior to the intervention.

Our case is unique in highlighting the possibility of cardiac tamponade in lcSSc, after prolonged remission within seven years of diagnosis. Our patient had PHTN, but no renal involvement. There is also an arguable possibility of COVID-19 infection triggering the flare-up of her lcSSc causing severe pericardial disease. Several infectious agents have already been speculated to trigger SSc; however, the role of COVID-19 infection has yet to be fully understood. Rimar et al. reported a case of scleroderma renal crisis post-COVID-19 infection in a patient with lcSSc after long remission [10]. This may further add to the concern of risk of severe disease in SSc after COVID-19 infection when taken together with findings in our patient.

## Conclusions

As discussed, pericardial disease can be silent in SSc. Even with large effusions, cardiac tamponade rarely develops. Considering findings from the literature and our patient’s case, it is reasonable to keep a high suspicion of pericardial tamponade in patients with a history of SSc, even lcSSc, who develop new heart failure or compromise; prompt echocardiographic screening can assist with diagnosis. Suspicion should also be high post-COVID-19 infections and close follow-ups with these patients may help recognize this life-threatening complication early.
